# Contextual Conversational Agent to Address Vaccine Hesitancy: Protocol for a Design-Based Research Study

**DOI:** 10.2196/38043

**Published:** 2022-08-23

**Authors:** Youness Zidoun, Sreelekshmi Kaladhara, Leigh Powell, Radwa Nour, Hanan Al Suwaidi, Nabil Zary

**Affiliations:** 1 Institute for Excellence in Health Professions Education Mohammed Bin Rashid University of Medicine and Health Sciences Dubai United Arab Emirates; 2 College of Medicine Mohammed Bin Rashid University of Medicine and Health Sciences Dubai United Arab Emirates

**Keywords:** conversational agent, design-based research, chatbot, Rasa, NLU, COVID-19, vaccine hesitancy, misinformation, vaccination, iterative design, health communication, health information, System Usability Scale

## Abstract

**Background:**

Since the beginning of the COVID-19 pandemic, people have been exposed to misinformation, leading to many myths about SARS-CoV-2 and the vaccines against it. As this situation does not seem to end soon, many authorities and health organizations, including the World Health Organization (WHO), are utilizing conversational agents (CAs) in their fight against it. Although the impact and usage of these novel digital strategies are noticeable, the design of the CAs remains key to their success.

**Objective:**

This study describes the use of design-based research (DBR) for contextual CA design to address vaccine hesitancy. In addition, this protocol will examine the impact of DBR on CA design to understand how this iterative process can enhance accuracy and performance.

**Methods:**

A DBR methodology will be used for this study. Each phase of analysis, design, and evaluation of each design cycle inform the next one via its outcomes. An anticipated generic strategy will be formed after completing the first iteration. Using multiple research studies, frameworks and theoretical approaches are tested and evaluated through the different design cycles. User perception of the CA will be analyzed or collected by implementing a usability assessment during every evaluation phase using the System Usability Scale. The PARADISE (PARAdigm for Dialogue System Evaluation) method will be adopted to calculate the performance of this text-based CA.

**Results:**

Two phases of the first design cycle (design and evaluation) were completed at the time of this writing (April 2022). The research team is currently reviewing the natural-language understanding model as part of the conversation-driven development (CDD) process in preparation for the first pilot intervention, which will conclude the CA’s first design cycle. In addition, conversational data will be analyzed quantitatively and qualitatively as part of the reflection and revision process to inform the subsequent design cycles. This project plans for three rounds of design cycles, resulting in various studies spreading outcomes and conclusions. The results of the first study describing the entire first design cycle are expected to be submitted for publication before the end of 2022.

**Conclusions:**

CAs constitute an innovative way of delivering health communication information. However, they are primarily used to contribute to behavioral change or educate people about health issues. Therefore, health chatbots’ impact should be carefully designed to meet outcomes. DBR can help shape a holistic understanding of the process of CA conception. This protocol describes the design of VWise, a contextual CA that aims to address vaccine hesitancy using the DBR methodology. The results of this study will help identify the strengths and flaws of DBR’s application to such innovative projects.

## Introduction

### Background

Conversational agents (CAs), sometimes known as chatbots, are an emerging technology for facilitating health communication. They are becoming increasingly popular in various professions, including education, entertainment, and health care. Furthermore, CAs are becoming more widespread in formal health care settings to aid with essential duties including appointment scheduling and health monitoring [1,2]. As technology advances, more advanced CAs that can engage humans in natural communication are beginning to appear. As a result, these technologies are becoming more common in daily health-related activities [3], medical care such as orthopedics [4], virtual medical consultations [5], pediatric care [1], geriatric care [6], public health and surveillance [7], large-scale monitoring systems [8], etc. CAs are typically deployed through a variety of platforms such as social networks (eg, Facebook and Twitter), messaging apps (eg, WhatsApp, Discord, and Telegram), or even emails and webpage widgets, making them highly accessible and sustainable [9].

Already deployed in the fight against the COVID-19 pandemic, many authorities and health organizations, including the World Health Organization (WHO) [2], the UK government [10], the US Department of Veterans [11], and the Saudi Ministry of Health [12], have embraced these emerging technologies. In addition, health CAs have been introduced as an innovative digital intervention to support the fight against the pandemic [13]. However, the efficiency of design and best practice for development remains a field of exploration for researchers. The effectiveness of a CA’s design remains key to its success, mainly when its purpose is to address serious concerns such as misinformation, mental health, or behavior change.

Many design approaches have been presented in the literature [14,15], ranging from early forms of hard-coded response generators to advanced artificial intelligence (AI) development methodologies. These can be divided into two categories: rule-based and neural network-based methodologies. A neural network is based on deep learning models, whereas a rule-based system is based on established templates and answers.

Most of these design strategies use ontologies, which are based on the domain’s knowledge base and may be utilized to interpret the user’s intentions and address the difficulty of interpreting the user’s phrases [16].

On the other hand, model-driven chatbot development is a different approach. It comprises a neutral meta-model and a domain-specific language for chatbot description, code generators and parsers for several chatbot platforms, and a platform recommender. This approach supports forward and reverse engineering and model-based analysis. The feasibility of this later is presented via a prototype tool and an evaluation based on migrating third-party Dialogflow bots to Rasa [17].

AIML, an XML derivative, is also a widely used approach. The purpose of the AIML language is to simplify the role of conversational modeling in relation to a “stimulus-response” process. It is also a mark-up language based on XML and depends on tags that are the identifiers that make snippets of codes to send commands into the Chatbot [18].

### Design-Based Research to Design a CA

**Figure 1:**
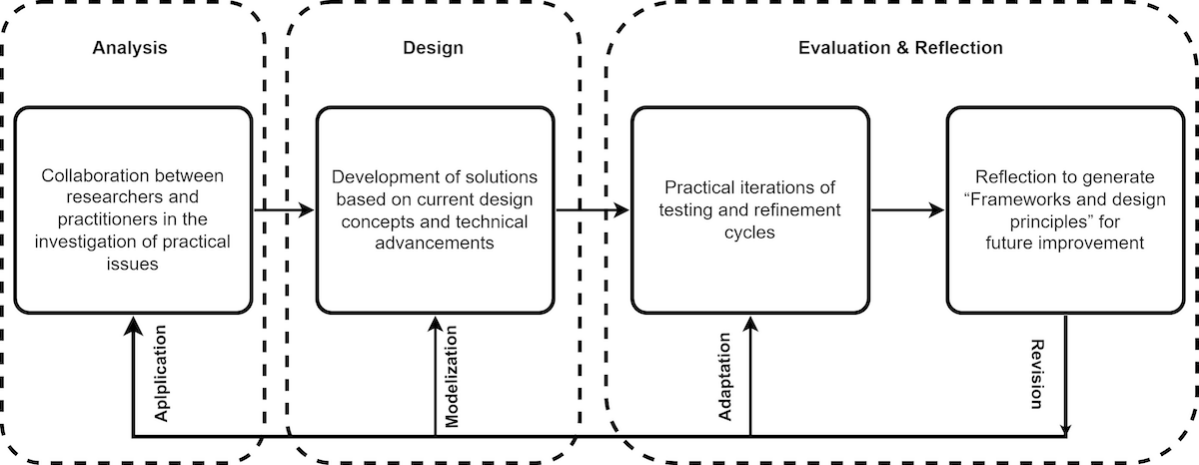
Design-based research approach adapted from Reeves [26].

Aligned with research methods from other fields in which products are developed for specific purposes [19-21], design-based research (DBR) is a methodological approach that relies on an iterative process. First, the problem that needs to be addressed is identified. Next, theoretical tools including models or frameworks are created to represent the potential solution to the problem. These are later tested in a real environment to measure their impact. As the testing progresses, the theoretical tools are assessed on the basis of new evidence of their positive or lack of effectiveness, and real-time revisions are made if needed [22]. Different ideas can be found in the literature regarding the definition of DBR; however, all agree that it is divided into many stages [23-25]. The most common way of picturing the DBR process is shown in Figure 1.

Evidence from the literature agrees that DBR is a long-term approach that includes multiple iterations of design, development, and evaluation [27,28]. However, this is not always applicable for short-time-frame projects. This highlights how DBR may be effectively translated and utilized in CA projects, especially how many cycles are necessary to develop valid and meaningful design principles in a very short-term project.

A team of researchers conducted a study using a DBR approach to promote self-direction training and problem-based learning (PBL) for medical students in a clinical context [29]. Another design-based study [18] was conducted with the primary objective of improving the professional performance of higher education teachers by considering various pedagogical aspects and the need for communication and alignment of pedagogy and assessment.

In another DBR, researchers conducted a study aiming to promote and strengthen clinical reasoning and competencies and develop essential clinical skills for nurses. The study’s primary aim and approach were to create and construct a web-based learning management system that could promote PBL to students and define their clinical goals [30].

Another study shows that DBR contributed to advancing the practice by encouraging collaboration and coordination between practice and theory [31].

The findings of all these studies and more [32-34] were mostly reported as a basis for reproducing such digital projects and interventions, which helped DBR to demonstrate its potential as a methodology suitable for research and design technology-enhanced learning environments.

### Objective

This study protocol describes the use of DBR methodology to conceive a CA that aims to address vaccine hesitancy through a contextualized conversation. Three complementary objectives will be considered in the CA design: (1) combat misconceptions through the dissemination of trustworthy information in accordance with the WHO’s frequently asked questions (FAQs) (the updated version of January 2022 from the WHO’s FAQs [35] as a baseline data set feeding the AI model of the CA), (2) user profiling (a vaccine hesitancy profile is defined for users through the conversation), and (3) redirection and dissemination (users can disseminate good information and advocate for vaccination literacy). We hypothesize that DBR methodology improves the usability and accuracy of CAs. This intervention will use validated instruments to assess key metrics and present data interpretation.

## Methods

### Overview

This study entails a pragmatic approach that focuses on the practical application of the DBR methodology. Pragmatism is closely connected with DBR [36] as it allows one to go deeper into a data set to comprehend its significance and utilize the experimental approach to validate conclusions with consideration to subsequent iterations of the design.

As a public health education intervention, VWise CA conception will be considered an educational innovation potentially compatible with all generic DBR frameworks that aim to help design such a technological tool.

### Study Phases

**Figure 2:**
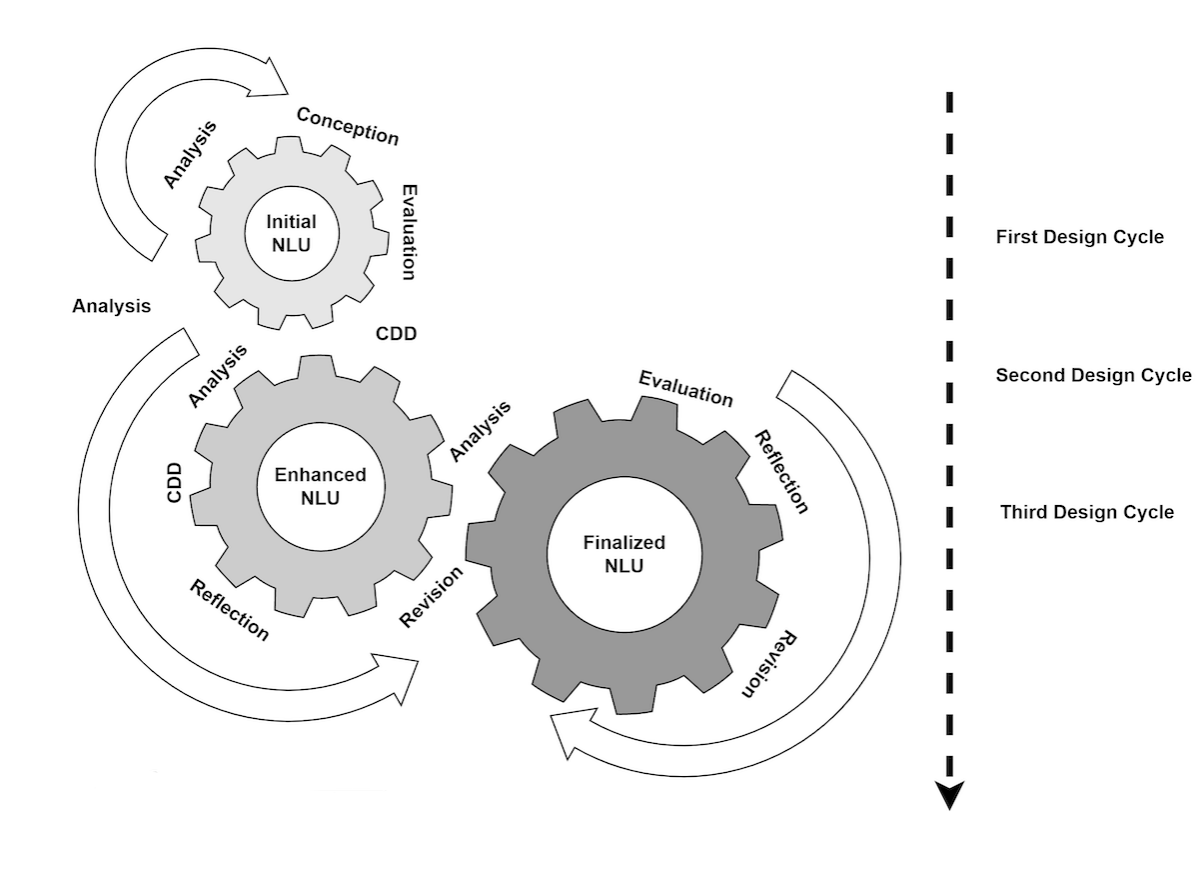
The study iterations cycles. CDD: conversation-driven development; NLU: natural-language understanding.

Aligned with DBR principles, this study will proceed in iterative cycles, as illustrated in Figure 2. The final objective is to finalize a natural-language understanding (NLU) model developed through cycles of analysis, design, evaluation, and reflection.

#### First Design Cycle: WHO-Based CA

Every complete cycle of design consists of three phases: analysis, design, and evaluation. In the first cycle, the analysis and exploration phase will begin with a need analysis establishing deficiencies of current public health education interventions related to vaccine hesitancy. Next, research questions are formulated, and a scoping review is conducted. The purpose at this stage is to understand the use of CAs in health education interventions. Another goal in the analysis phase is to identify the different aspects considered during the design.

In the design phase, the initial NLU model based on the WHO’s FAQs data set is developed and integrated using Rasa, an open-source platform for building CAs. During this phase, FAQs are mapped into intents and utters, and the development team creates a set of initial training data to build the first NLU prototype.

**Figure 3:**
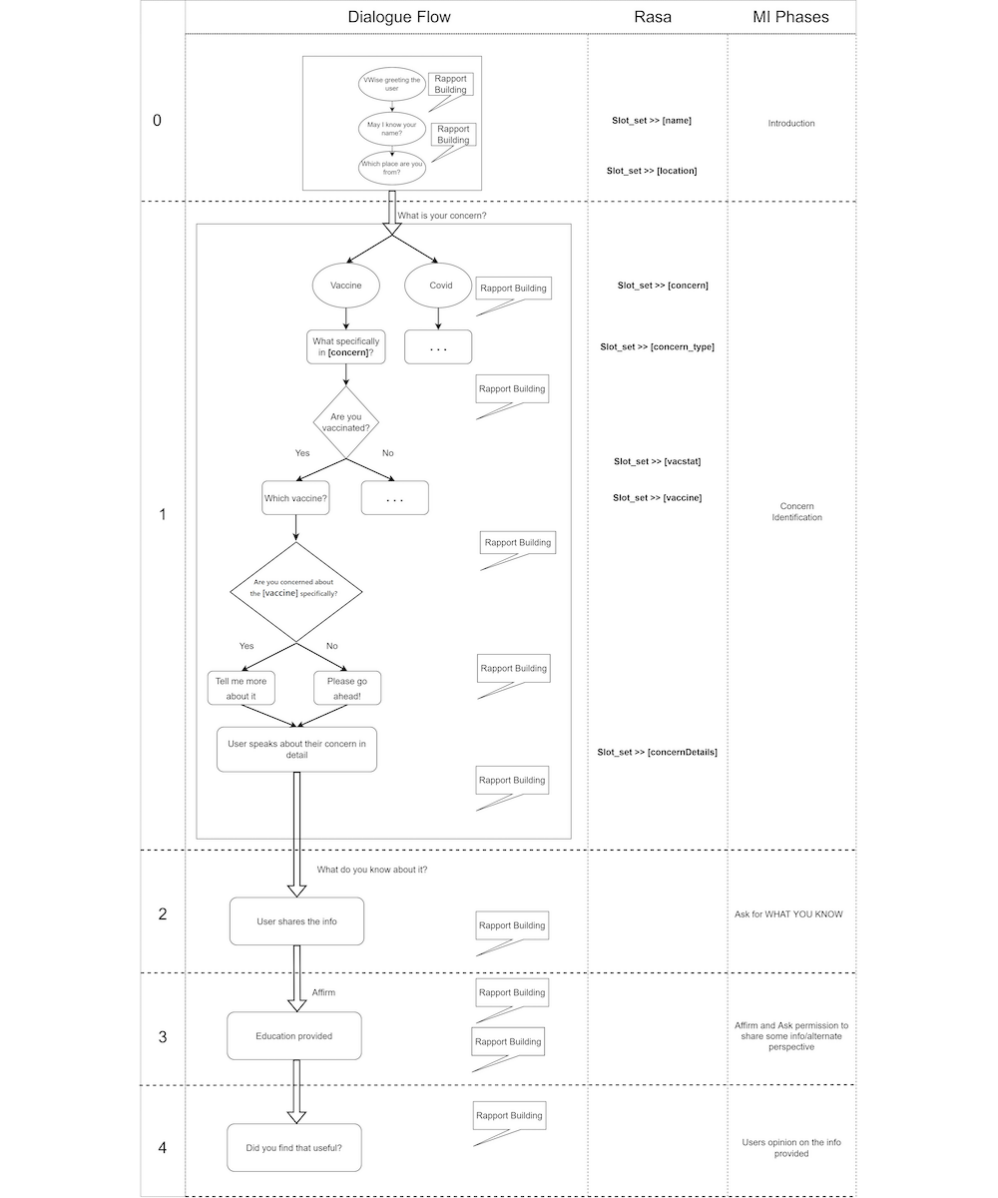
Conversation framework based on Motivational Interviewing (MI) techniques.

Conversational design (CD) will then be implemented to engage the users in the conversation. This helps to elevate the bot from an FAQ level to a more conversational context. The CD part of the CA is based on the Motivational Interviewing (MI) phases [37]. Figure 3 shows the conversation framework based on the MI phases.

A dialogue map defines various dialogue stages that constitute the conversation flow in each MI phase. Each phase can be divided into sub- or microphases (1.1, 1.2, etc). Rasa Map defines the technical aspect of, for example, how a concern is identified and stored in a database for analysis at a later stage.

The first step is bot introduction, where greeting and collection of user demographics (Vaccine status, age, and gender) occur. Concern identification is a crucial part of the whole process determining the framework’s branching. Then, the user’s knowledge is challenged to establish the level of awareness and readiness for the educational part. Education is thereby provided in accordance with previous data collected. Finally, opinion about the new information presented is gathered at the latest stage.

Best practices from Rasa documentation [38] are also considered in this phase. This means that remarks and observations made from a project management perspective during the analysis phase are encapsulated in the initial model. This basic chatbot is then tested within the team (team members who were not part of the bot’s development to avoid biasing) in 5 testing rounds, each being a microcycle in the design phase.

The evaluation and reflection phase consists of several internal testing cycles. In addition, a conversation-driven development (CDD) process, which is a method that encourages listening to users and using their insights to improve the AI assistant, is also initiated at this stage [39]. As an overarching best practice approach for chatbot development, the CDD approach includes the following actions: (1) we will share the assistant with users as soon as possible, (2) we will review conversations regularly, (3) we will annotate messages and use them as NLU training data, (4) we will test that the assistant always behaves as expected, (5) we will track when the assistant fails and measure its performance over time, and (6) we will fix how the assistant handles unsuccessful conversations.

**Figure 4:**
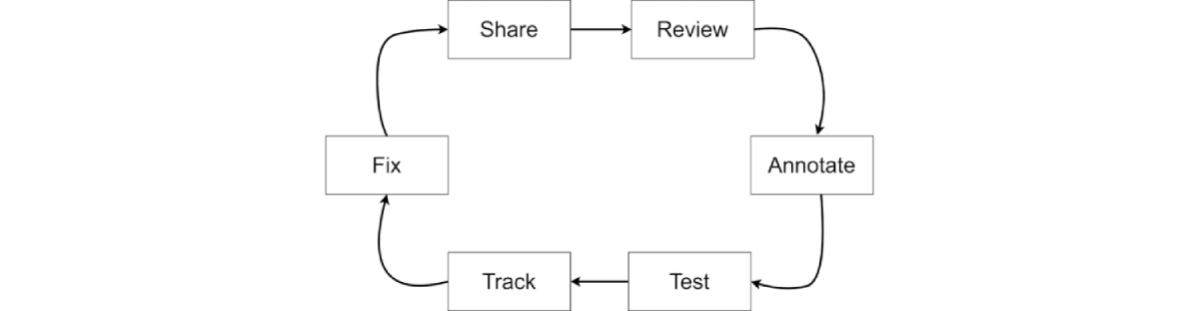
The iterative conversation-driven development (CDD) process.

As illustrated in Figure 4, the CDD method is not a linear process; the chatbot designer should iterate repeatedly as the development and improvement of the bot are needed.

The System Usability Scale (SUS) will be adopted in the reflection phase since it is a well-established usability instrument that has been used repeatedly [40,41]. It should be noticed that this is not a standardized way of assessing CAs, as in many cases, a single survey question was utilized as an alternative [30]. Unlike evaluating the CA performance in terms of usability, performance evaluation will also improve the intent-utter predictions. First, we shall calculate the conversations’ precision, recall, and F1-score by establishing a confusion matrix of intents. The three measurements are defined as follows:

Precision (P) = (True positives) / (True positives + False positives)

Recall (R) = (True positives) / (True positives + False negatives)

F1-score = 2 × [(P × R) / (P + R)]

Second, the PARADISE (PARAdigm for Dialogue System Evaluation) framework [29] will provide better conversational value to the CA.

This method uses a decision-theoretic framework to specify the relative contribution of various factors to an agent’s overall performance. The method states that the performance of a chatbot can be associated with the external criteria (user satisfaction), which is the goal of chatbot development. Task success and dialogue costs are the two factors that contribute to achieving this goal. The κ coefficient is used to understand the task success. Cost measures considered in PARADISE reflect both naturalness and efficiency of a chatbot’s behavior.

An attribute value matrix is constructed, which represents the dialogue tasks. This consists of the information that must be exchanged between the agent and the user during the dialogue. It is represented as a set of ordered pairs of attributes and their possible values.

Performance evaluation for an agent requires a corpus of dialogues between users and the agent, in which users execute a set of scenarios. κ is calculated from a confusion matrix that summarizes how well an agent achieves the information requirements of a particular task for a set of dialogues instantiating a set of scenarios.

The values in the matrix cells are based on queries and their corresponding responses by the CA. For a particular user query, if the predicted response matches the intended response, the number in the appropriate diagonal cell of the matrix is incremented by 1. The off-diagonal cells represent misunderstandings that are not corrected in the dialogue. For example, “mourn” could be confused with “morning.” The effect of misunderstandings that are corrected during the dialogue is reflected in the costs associated with the dialogue. An example of a confusion matrix built on a subset of intents is shown in Table 1.

**Table 1:** An example of the confusion matrix.

Intents	greet	Side-effect	Vaccine safety	mRNA	affirm	Recall	Precision	F1-score
greet	10	—^a^	—	—	1	0.091	0.909	0.165
Side-effect	—	13	7	—	—	0.65	0.867	0.743
Vaccine safety	—	2	5	—	—	0.714	0.417	0.527
mRNA	—	—	—	8	—	1	1	1
affirm	1	—	—	—	9	0.9	0.9	0.9

^a^—: Not applicable.

Columns represent the key, specifying which information values the agent and user were supposed to communicate to one another given a particular scenario. Rows represent the data collected from the dialogue corpus (final NLU), reflecting what attribute values were communicated between the agent and the user.

In a confusion matrix M, success in meeting the information requirements of the task is measured by κ [42]:

κ = [P (A) – P (E)] / [1 – P (E)]

P(A) is the proportion of times the actual answer matches the prediction. P(E) is the proportion of times the actual response and prediction should coincide by chance. The value of κ is always between 0 and 1. If no random match is expected, κ would be 0. If there is a complete match, κ=1 [43].

#### Second Design Cycle: Subject Matter Expert–Based CA

As explained previously, DBR is an iterative process; therefore, the three phases of analysis, design, and reflection are continuously repeated in each design cycle. In addition to the results of the preceding cycle, the new input to this new cycle of analysis and exploration will be the intervention of subject matter experts as part of the CDD process. They will analyze, review, annotate, and correct the previous NLU model through conversations data that will result from the previous iteration. Errors and imperfections will be flagged and annotated for the technical team to adjust and incorporate during the new design cycle. A revised NLU model will be generated, trained, and then integrated to fuel a new pilot round that will provide data for reflection and revision. All evaluation metrics used during the first design cycle will be repeated to compare the evolution of the CA design.

#### Third Design Cycle: Final CA

To complete the third iteration of the design cycles, the three phases will again be conducted, but this time as a consolidation iteration that explores previous flaws, mismatches, and analyses recurring errors and identify deep issues in the prior design of the NLU model. The correction will then be implemented and tested for compliance and alignment with the final expected outcomes. Thus, the last pilot will be carried out as part of the evaluation and reflection phase to ensure the reliability of the NLU model and conclude the design cycles to implement the CA for a larger group of participants via social media platforms.

### Data Collection

This study will utilize a convenience sampling approach to recruit participants. Since no sample size recommendation is noticeable in the literature as data sufficiency, a sample size of 500 participants is expected throughout the entire design iterations. New participants are needed for each iteration in accordance with Rasa’s best practice for building CAs. Data will be collected via a web server hosted locally at Mohammed Bin Rashid University of Medicine and Health Sciences (MBRU), and only the research team will be accessing data. Participants will be prompted to conversate with the chatbot directly. First, a consent form is completed, and then the conversation begins in accordance with the dialog flow designed. Demographic data such as age, gender, nationality, and vaccination status will be collected from participants anonymously. Concerns and specific knowledge are then discussed, as well as readiness to receive new information.

Users will be invited to interact with the chatbot as much as they want with only a notice that they must respect a minimum of 10 minutes to be considered valid entry data to the analysis. They can ask questions about COVID-19 and vaccination in their own words. Participants will then be asked about their experience with the chatbot using the SUS.

### Ethical Approval

The research protocol was approved by the Ethical Review Board of MBRU, located in Dubai, United Arab Emirates, in October 2021 [Approval no MBRU IRB-2021-67].

### Data Analysis

Conversation data are reviewed at each evaluation iteration through the Rasa X graphical user interface to improve the NLU model on the basis of messages from the same real conversations. Annotating user messages is a great way to understand the bot’s successes and failures and add data that reflect what real users are saying to the assistant. The Insights section in Rasa X will help understand how to improve NLU training data. It will also provide various suggestions for the messages in the NLU inbox.

Among other things, Rasa X uses cross-validation results to identify problems in the training data. It uses 4-fold cross-validation, meaning that it trains four models on 80% of the training data and evaluates them on the remaining 20%. A minimum number of examples calculator ensures that each intent has at least this number of examples (20 by default); otherwise, a new insight is created [44].

When reviewing conversations, tags are created to track what happened in conversations and mark specific messages to pinpoint problems. In addition, these tags can filter conversations to help find successful and unsuccessful conversations.

Using the examples provided as training data, a confidence score is computed for each user’s intent. The confidence value is computed as a similarity index after deriving appropriate features embedded in a high-dimensional plane. The response corresponding to a higher confidence value is selected as the response from CA to the user intent. The confidence values for each user query and intent are available in the tracker memory and can be written to a database for further analysis.

To measure users’ satisfaction with VWise, usability will be evaluated after the testing phases of each design cycle using SUS. As a nonproprietary and technology-agnostic metric, this user experience assessment will help inform every new design cycle for quality improvement. The precision, recall, and F1-score will be calculated through the confusion matrix, giving insight into how well VWise is achieving the information requirements of a task for a set of dialogues. The dialogue success will be captured later from κ as part of the PARADISE paradigm that will be adopted carefully to the application of this study [42].

## Results

As of this writing (April 2022), an initial NLU model was designed. The research team reviews the model as part of the CDD process, preparing it for the first pilot that will wrap up the first design cycle of the CA. Conversational data collected via Rasa X will be analyzed quantitatively and qualitatively (see *Methods*) to inform the reflection and revision phase. Three iterations are identified in this project, resulting in several studies disseminating findings and conclusions. The first study describing the full initial design cycle will be submitted for publication before the end of 2022.

We aim to report all findings in a final scientific paper describing the results of using DBR to conceive a CA that profiles participants on the basis of their hesitancy to get vaccinated and addresses their concerns by the end of the three design cycles.

## Discussion

### Expected Findings

This research examines the hypothesis that DBR can improve the accuracy and performance of a CA.

This protocol describes the research and design methods and processes to develop a contextual CA to address, profile, and inform about vaccine hesitancy. In addition, this study protocol contributes to understanding how DBR can drive chatbot design and conception and enhance its performance for public health education interventions starting from FAQ data sets and AI best practices.

This protocol needed researchers from different disciplines to establish a common ground for combining machine learning mechanisms and some learning design technics during designing cycles. In addition, some DBR principles and models were expanded or adapted to clarify the overall design process and how to perform such research with the support of this methodology [24].

The perspectives of AI, public health education, and learning design are combined to generate insight into vaccine hesitancy attitudes and behaviors when confronted with CAs as health education tools. This resulted in the approach that combines these perspectives inside a generic iterative methodology that can inform similar research interventions.

Owing to its holistic approach, this protocol is particularly valuable. In this approach, the respect for the three iterative phases in a design cycle plays an essential role in generating the final NLU model. The more traditional ways of applying DBR are often too generic to accommodate the design of a health education tool such as the VWise agent.

A more generic strategy is provided to start the following design cycle process by complementing the first iteration of the designing cycle. The adaptation and expansion of such a methodology have been discussed before in the literature [24,27,45]. For example, it is emphasized that it is required to build on the generic concept of DBR as traditionally perceived by addressing the process’s subcomponents in terms of different-sized cycles, notably micro-, meso-, and macrocycles [24,45].

This research can bring together multidisciplinary teams to understand the CA design process. Furthermore, this novel approach will help establish DBR in the AI environment, bridging engineering principles and research methodologies to serve different applications that can benefit multiple fields.

Owing to the multidisciplinary nature of this research project, we hope that our efforts to describe the research approach will contribute to the reliability and reproducibility of this type of research, particularly in public health education.

### Limitations

One of the limitations of the protocol is the convenience sampling approach utilized in the pilot studies during the evaluation phases.

While convenience sampling could potentially introduce bias to the study results [46], there is another problem that is of great concern in this context—outliers. Because of the high possibility of self-selection in nonprobability sampling, the effects of outliers can be even more devastating in this type of subject selection. Outliers are cases that are considered not to belong to the data. In a random sample, however, neither outliers nor their probabilities are quantified [47]. The researcher does not know how well a random sample represents the population in terms of the characteristics or mechanisms being studied. What makes samples so unpredictable is their susceptibility to serious hidden biases [48].

On the other hand, this method is still a practical approach used by various studies, especially in the absence of a sampling frame. However, we expect this to be a minor issue of the study as this phase aims to test the NLU models only, and all revisions will be based on the researchers’ consensus regarding the best way to correct and address flaws. Another limitation is the lack of models and frameworks that can guide this approach as a reference to DBR application to the CA’s field.

### Conclusions

Developing a highly accurate CA is vital to delivering any behavioral health outcomes. To develop a CA that can significantly impact vaccine hesitancy, it is crucial to address real users’ needs that are exposed daily to an enormous amount of misinformation, offer consistent and reliable data to gain trust and credibility, and most importantly, adapt and contextualize responses. DBR can enhance chatbot performances by providing a pragmatic framework for continuous development and iterative correction. This study will not only offer researchers a validated methodology for designing a CA, but it will also provide a path for scaling public health education projects.

## Abbreviations

AI: artificial intelligence
CA: conversational agent
CD: conversational design
CDD: conversation-driven design
DBR: design-based research
FAQ: frequently asked question
MBRU: Mohammed Bin Rashid University of Medicine and Health Sciences
MI: motivational interviewing
NLU: natural-language understanding
PARADISE: PARAdigm for Dialogue System Evaluation
PBL: problem-based learning
SUS: System Usability Scale
WHO: World Health Organization

## Data Availability

Supporting data cannot be made public owing to ethical concerns. Upon reasonable request to the corresponding author and after the study’s findings have been published, data may also be made available.
